# Predictive value of admission blood glucose for early severity stratification in pediatric scorpion envenomation

**DOI:** 10.1590/1678-9199-JVATITD-2025-0074

**Published:** 2026-03-06

**Authors:** Tatiane Felícia dos Santos, Ana Thereza Chaves Lages, Adebal Andrade, João Saldanha, Gisele Gomes da Silva, Carlos Chavez-Olortegui, Manoel Otávio da Costa Rocha

**Affiliations:** 1 Graduate Program in Health Sciences - Infectious Diseases and Tropical Medicine, Medical School, Federal University of Minas Gerais (UFMG), Belo Horizonte, MG, Brazil.; 2 Urgency and Emergency Hospital Complex, João XXIII Hospital, Belo Horizonte, MG, Brazil.; 3 Department of Toxicology, Urgency and Emergency Hospital Complex, João XXIII Hospital, Belo Horizonte, MG, Brazil.; 4 Toxicological Information and Assistance Center of Minas Gerais (CIAToxMG), Belo Horizonte, MG, Brazil.; 5 Department of Biochemistry and Immunology, Institute of Biological Sciences (ICB), Federal University of Minas Gerais (UFMG), Belo Horizonte, MG, Brazil.

**Keywords:** Scorpion stings, Envenomation, Hyperglycemia, Public health, Tityus serrulatus

## Abstract

**Background:**

Scorpion stings are an increasing public health concern in Brazil, where children are at a heightened risk of severe outcomes. Simple biomarkers, such as admission blood glucose may improve early risk stratification. To assess the association between admission blood glucose and clinical severity in children envenomated by *Tityus serrulatus*.

**Methods:**

This prospective observational cohort study was conducted at João XXIII Hospital, Minas Gerais State Hospital Foundation (FHEMIG), Belo Horizonte, Brazil, from September 2023 to March 2024. We included patients aged 0 to 17 years with confirmed scorpion envenomation. Clinical severity was classified as mild (no antivenom) or moderate/severe (antivenom administered). Blood glucose was measured at admission and two hours thereafter. Predictive performance was assessed using receiver operating characteristic (ROC) analysis.

**Results:**

Sixty-seven children were included; 13.4% progressed to moderate/severe envenomation. Admission glucose ≥ 142 mg/dL showed 100% specificity and a 100% positive predictive value for severe cases (ROC AUC 0.979; 95% CI, 0.937-1.000; p < 0.001). A 105 mg/dL threshold maximized sensitivity (100%) at the expense of lower specificity (79.3%). To our knowledge, this is the first study to systematically evaluate admission glucose in pediatric *T. serrulatus* envenomation and to propose cutoffs for risk stratification.

**Conclusion:**

Admission blood glucose is an accessible and accurate biomarker for early triage of severity in pediatric scorpion envenomation. A 105 mg/dL threshold favors maximal sensitivity for screening, whereas 142 mg/dL ensures high specificity for severe cases. Although promising, these cutoffs should be treated as clinical hypotheses and require multicenter external validation before widespread adoption.

## Visual Abstract

**Figure:**
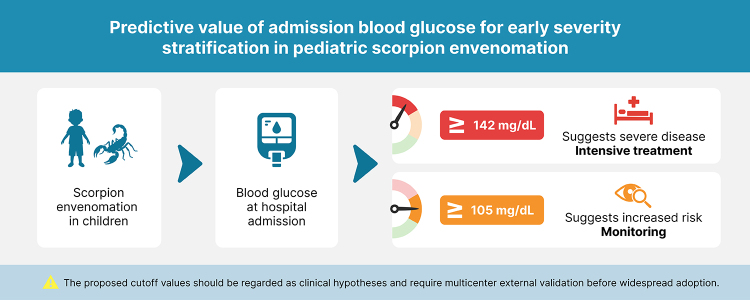
Visual Abstract

## Background

Scorpion stings are the primary cause of envenomation by venomous animals in Brazil and their incidence has been progressively increasing, with a higher risk of morbidity and mortality in children [1]. In 2022, the fatality rate was 0.58%, concentrated in children under 14 years of age [2]. 

This condition represents a significant public health challenge [3], with children and the elderly being the most vulnerable due to immunological immaturity, immunosenescence, and, in the case of children, a lower body mass [4, 5]. Various factors influence clinical severity, such as the age, the species involved, the amount of venom, the sting site, the time to treatment, and the use of antivenom [6-8]. Among the clinical manifestations, hyperglycemia has been frequently described in moderate to severe cases, although the magnitude of this association remains to be fully defined [9-17].

Given the scarcity of detailed clinical data, especially involving *Tityus serrulatus*, this study sought to evaluate the relationship between blood glucose at hospital admission and morbidity in children treated at a toxicology reference center in Minas Gerais, Brazil, thereby contributing to risk stratification strategies and the reduction of morbidity and mortality.

In this study, we hypothesized that admission blood glucose may function as an early diagnostic marker of severity in pediatric scorpion envenomation caused by *T. serrulatus*. Given that the venom of this species triggers an intense and rapid adrenergic surge capable of inducing hyperglycemia even before full clinical manifestations arise, we propose that elevated glucose levels may signal an increased risk of progression to moderate or severe envenomation. Accordingly, we assessed the discriminatory performance of admission glucose as a tool for early risk stratification, offering a simple, accessible, and potentially useful biomarker for the initial triage of children presenting with scorpion stings.

## Methods

This short-term prospective observational study was conducted from September 2023 to March 2024 at João XXIII Hospital (FHEMIG), in Belo Horizonte, Minas Gerais, Brazil. The protocol was approved by the Research Ethics Committees of FHEMIG and the Federal University of Minas Gerais (UFMG) (CAAE: 67636623.7.3002.5119 and 67636623.7.0000.5149). Written informed consent was obtained from participants’ legal guardians.

The inclusion criteria were: children and adolescents aged 0 to 17 years, of both sexes, with confirmed *T. serrulatus* stings (visual identification or the specimen brought to the hospital). The exclusion criteria were: cases in which the species could not be confirmed, admission occurring more than six hours after the sting, prior treatment in another unit before transfer, or a previous diagnosis of diabetes mellitus.

Blood glucose was measured at admission and two hours later, in both patients treated with antivenom and those under observation only. Cases were classified as mild (no antivenom, observation, or analgesia only) or moderate/severe (treated with antivenom), following the Brazilian Ministry of Health protocols.

Severity was dichotomized (mild vs. moderate/severe). Numerical variables were described using measures of central tendency and dispersion and were compared using the Mann-Whitney U test; categorical variables were expressed as frequencies and compared using the chi-square or Fisher’s exact test. Variables with p < 0.20 were included in a multiple logistic regression model. Diagnostic accuracy was assessed using ROC curves, considering sensitivity, specificity, predictive values, and the Youden index. Analyses were performed using SPSS version 23, with a 5% significance level.

## Results

The study included 67 children, with an average age of 7.9 years; the majority were female (53.7%), of mixed race (73.1%), and lived in Belo Horizonte (50.7%). Accidents occurred primarily at home (91%), with patients arriving via their own transportation (80.6%). More than half were classified as “yellow” priority under the Manchester protocol (53.7%).

Stings occurred primarily on the lower limbs (43.3%), with *T. serrulatus* identified in 70.3% of cases. The most common manifestations were localized pain (98.5%), sweating (19.4%), paresthesia (11.9%), and uncontrollable vomiting (10.4%).

Nine children (13.4%) developed moderate or severe cases: three received two vials of antivenom and six received four. Two required intensive care and seven remained in the ward. The average length of stay was 8.4 hours in mild cases and 44.2 hours in moderate/severe cases.

### Comparative analysis of blood glucose in moderate/severe (treated) cases

**Figure 1.**
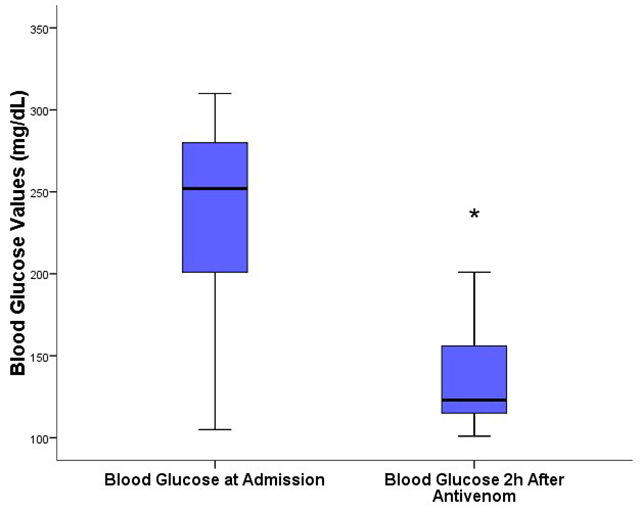
Blood glucose measurements upon hospital admission and two hours after antivenom administration. The graph represents the patients’ blood glucose levels at risk classification (box plot for “admission blood glucose”) and two hours after antivenom (SAE) administration (box plot for “2h post-antivenom blood glucose”). The data distribution is represented by five values in the diagram: central value (median), lower and upper sides of the box (first quartile 25% and third quartile 75%), and two extreme values (upper and lower limits). The asterisk indicates a significant difference at the 0.05 significance level. *Significant reduction (p = 0.008) in blood glucose levels two hours after antivenom administration compared to blood glucose levels upon hospital admission.

In the nine children classified as moderate/severe cases, blood glucose was measured upon admission, during risk screening, and two hours after antivenom administration. A significant reduction was observed (p = 0.008), with the median falling from 252 mg/dL to 123 mg/dL, a reduction of approximately half (Table 1; Figure 1).

**Table 1.** Comparison of blood glucose levels at admission and two hours after antivenom administration in patients with moderate/severe envenomation (n = 9).

Variable	Admission (n = 9)	2h after antivenom (n = 9)	p-value
Blood glucose	252 mg/dL (183-292 mg/dL)	123 mg/dL (2,4 pt)(109-171 mg/dL)	0.008

Median (IQR): Wilcoxon signed-rank test (Z = -2.666); p = 0.008.

IQR: interquartile range; n: number; mg: milligrams; dL: deciliters.

### Comparative analysis of blood glucose in mild (untreated) cases

**Figure 2.**
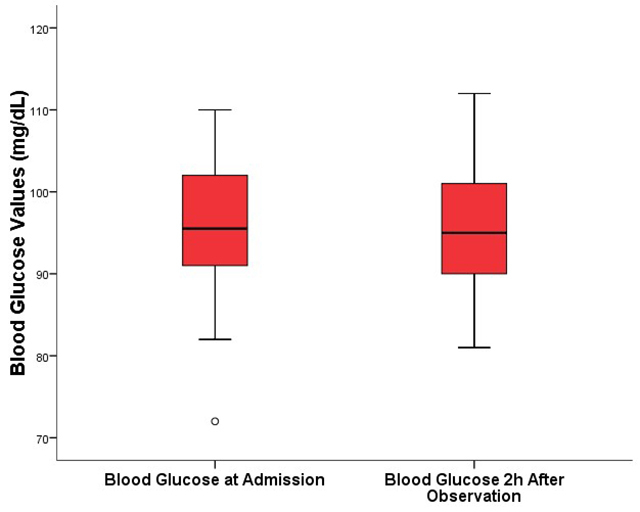
Blood glucose measurements upon hospital admission and two hours after observation. The graph represents the patients’ blood glucose levels at risk classification (box plot for “admission blood glucose”) and two hours after observation (box plot for “2h post-observation blood glucose”). The data distribution is represented by five values in the diagram: central value (median), lower and upper sides of the box (first quartile 25% and third quartile 75%), and two extreme values (upper and lower limits). No significant differences were observed between the evaluated times (p = 0.335). The small circle (◦) represents outlier values.

In the 58 children classified as mild cases, blood glucose was measured upon admission and two hours after observation. There was no significant reduction (p = 0.335), with the median ranging from 95.5 mg/dL to 95 mg/dL, remaining practically stable (Table 2; Figure 2).

**Table 2.** Comparison of blood glucose levels at admission and two hours after observation in patients with mild envenomation (n = 58).

Variable	Admission (n = 58)	2h after observation (n = 58)	p-value
Blood glucose	95.5 mg/dL (2,4 pt)(91-102 mg/dL)	95.0 mg/dL (2,4 pt)(90-102 mg/dL)	0.335

Median (IQR): Wilcoxon signed-rank test (Z = -0.964); p = 0.335.

IQR: interquartile range; n: number; mg: milligrams; dL: deciliters.

### Comparative analysis of variables between groups

Significant differences between severity groups were found for weight (p = 0.021), blood glucose (p < 0.001), Glasgow Coma Scale (p < 0.001), respiratory rate (p < 0.001), uncontrollable vomiting (p < 0.001), sweating (p = 0.001), intense sweating (p = 0.001), prostration (p = 0.016), and sensory depression (p = 0.016). Children who developed severe cases had lower weight - up to half the median of the mild group - and tended to be younger, although age did not reach statistical significance. Elevated blood glucose and respiratory rate emerged as key severity markers. Clinically, uncontrollable vomiting, intense sweating, prostration, and sensory depression were exclusive to severe cases, while sweating was more frequent.

### Evaluation of blood glucose diagnostic capacity

**Figure 3.**
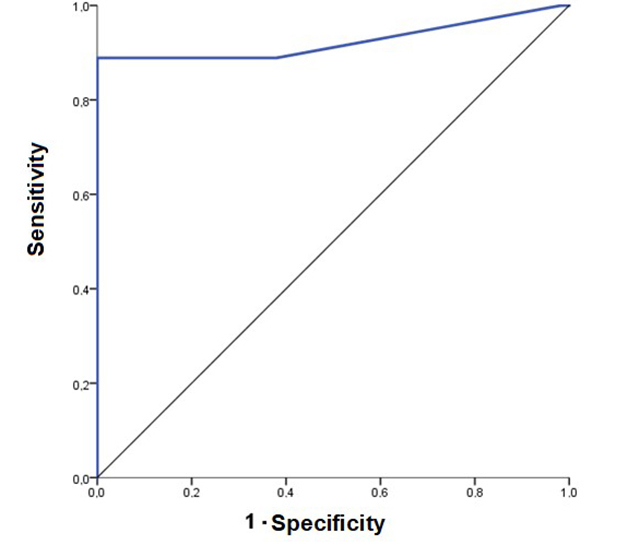
Accuracy of blood glucose in diagnosing moderate/severe scorpionism cases. The graph represents sensitivity on the Y-axis and specificity on the X-axis (in the form of 1-specificity). The Y-point is high on the axis, indicating high sensitivity, and the X-point is near the origin, also showing high specificity. The obtained accuracy was significant (p < 0.001).

The accuracy of blood glucose as a severity marker was high, with an AUC of 0.979. Table 3 summarizes the values, and Figure 3 shows the ROC curve, illustrating diagnostic performance at different cutoff points.

**Table 3.** Accuracy of blood glucose as a diagnostic marker for moderate/severe scorpionism cases.

Area	p-value	95%CI
0.979	< 0.001	0.937-1.000

CI: confidence interval.

Blood glucose showed high diagnostic accuracy (AUC = 0.979; p < 0.001), correctly classifying 97.9% of cases (95%CI: 0.937-1.000). The best cutoff was 142 mg/dL, with 89% sensitivity, 100% specificity, a PPV of 100% and a NPV of 98.3%. For screening purposes, 105 mg/dL was also effective, offering 100% sensitivity but lower specificity (79.3%).

## Discussion

Hyperglycemia has been repeatedly associated with greater severity of scorpion envenomation in children [18]. In the present study, blood glucose functioned as a useful severity marker: all moderate/severe cases presented hyperglycemia at admission, and a significant reduction was observed two hours after antivenom administration, although values remained above the normal range - findings consistent with the therapeutic effect of antivenom.

In scorpion envenomation, hyperglycemia appears to be mediated predominantly by an autonomic storm characterized by brisk catecholamine outflow and additional counterregulatory hormones, a pathway repeatedly documented and associated with worse clinical outcomes [14]. In *T. serrulatus*, venom composition is dominated by neurotoxins that act on ion channels (NaV/Kv), which underpins the observed systemic phenotype [19]. In murine models, *T. serrulatus* envenomation induces a rapid increase in blood glucose with a concomitant insulin drop mediated by the pancreatic IL-1R/NO axis, and this response is exacerbated in the setting of pre-existing dysglycemia, reinforcing the translational relevance of glycemic markers for clinical risk stratification [20]. For other clinically important species, the endocrine-metabolic axis is likewise prominent but not identical: in *Mesobuthus tamulus* (India), the classical insulin-glucose infusion as adjunctive therapy emerged from observations of cardiometabolic dysfunction with marked hyperglycemia; in *Androctonus australis*, hyperglycemia is associated with adipose-tissue inflammation and TNF-α-mediated insulin resistance; and in *Centruroides* spp., experimental hyperglycemic responses have been documented [21, 22]. In this comparative context, it is plausible that optimal plasma-glucose cutoffs are not universal but vary by species and, potentially, by regional factors (venom composition and inoculated dose).

Literature reports show that blood glucose > 270 mg/dL is linked to heart failure and poor outcomes in pediatric scorpion envenomation in Tunisia [10]. Other studies have shown that admission hyperglycemia > 180 mg/dL is associated with respiratory failure, pulmonary edema, longer ICU length of stay, and increased mortality [23]; similarly, hyperglycemic children exhibit higher rates of shock, coma, heart failure, and pulmonary edema, with mortality disproportionately concentrated in the hyperglycemic group [18].

In this study, no hyperglycemia was detected in mild cases, even after two hours of observation, while severe cases showed endocrine changes consistent with a stronger systemic response. A cutoff of 142 mg/dL at admission provided the best specificity and PPV (100%), accurately excluding those not at risk and confirming severity in all above this level. The NPV was 98.3%, reinforcing reliability for negative cases. Literature on stress-induced hyperglycemia in scorpion envenomation is limited, with reported cutoffs ranging from 140-200 mg/dL and recommended targets between 110-150 mg/dL [24, 25].

Our primary contribution is to propose, for the first time, a plasma glucose cutoff of ≥ 142 mg/dL with 100% specificity and positive predictive value (PPV) for severe cases, providing an objective criterion that is immediately applicable to triage and initial management.

The findings of this study reinforce the idea that admission blood glucose can be used not merely as a complementary parameter but as a physiopathology-based triage tool capable of supporting early risk stratification in children stung by *T. serrulatus.* The magnitude of hyperglycemia observed exclusively in moderate/severe cases, as well as its significant reduction two hours after antivenom administration, suggests that the metabolic response is closely linked to the intensity of the autonomic storm induced by the venom. This temporal, physiological, and clinical coherence strengthens the proposal of using blood glucose as a marker of severity.

The clinical utility of this finding lies in the fact that many children may deteriorate within the first hours, and clinical signs of severity - such as intractable vomiting, prostration, or tachypnea - are not always present upon arrival. Elevated glucose thus emerges as an immediate, measurable indicator capable of alerting the triage team to the need for intensified monitoring, risk reclassification, or expedited medical evaluation. This potential is particularly relevant in Brazil, where *T. serrulatus* accounts for most severe cases, and in healthcare settings with high demand and limited resources.

Moreover, because the neurotoxic venom of *T. serrulatus* induces hyperactivation of ion channels and massive catecholamine release, the hyperglycemia associated with scorpion envenomation is not a nonspecific marker of stress but rather a metabolic signature of the venom’s systemic effects, with biological plausibility directly linked to its mechanism of action.

This aligns with evidence showing that stress-related hyperglycemia reflects systemic metabolic dysregulation in critical illness and may hold prognostic value in early risk assessment [26]. This supports the notion that a simple biomarker such as capillary glucose, can reflect the intensity of envenomation even before full clinical manifestations develop.

At admission, blood glucose ≥ 105 mg/dL demonstrated 100% sensitivity - minimizing false negatives but increasing false positives - highlighting the need for further studies to refine the optimal cutoff. Accordingly, the thresholds identified in this study - particularly 105 mg/dL (maximal sensitivity) and ≥ 142 mg/dL (100% specificity and PPV) - should not be interpreted as definitive clinical recommendations but as testable diagnostic hypotheses that may guide future research and encourage the use of glucose as a complementary triage tool in pediatric emergency care. Larger, multicenter studies will be needed to confirm or refine these thresholds, assess their performance across different regions, and determine their impact on clinical outcomes, including time to antivenom administration and need for intensive care.

Overall, 86.6% of cases were mild, 6% moderate, and 7.4% severe, consistent with literature showing predominance of mild cases and discharge without antivenom [27, 28]. Clinical characteristics also aligned with previous reports [29, 30]. Factors associated with progression to moderate/severe cases included lower weight, hyperglycemia, tachypnea, persistent vomiting, intense sweating, prostration, and decreased consciousness. Children under five years were disproportionately affected, confirming their greater vulnerability to severe outcomes [31, 32]. Because none of the patients showed clinical features suggestive of acute pancreatitis, an extended discussion of this complication was not included.

Children aged 0 to 14 years are more vulnerable to severe complications and death after scorpion stings due to smaller body size and immature physiological reserves, which amplify venom effects [33, 34]. In this study, tachypnea, persistent vomiting, and hyperglycemia at admission emerged as red flags for severe progression, underscoring the need for immediate triage and prompt initiation of care. Shortening the interval between risk classification and treatment is decisive for prognosis.

Although most patients arrived within two hours, more than half were triaged as yellow priority, which can delay antivenom administration and monitoring, worsening outcomes. Time is critical: each additional hour after the sting increases the risk of death by about 9% [27]. Mild cases generally arrived sooner than severe ones, often because the latter involved inter-municipal transfers. The mean length of stay was 8 hours for mild cases and 44 hours for severe cases (range: 2 hours to 6 days), with all patients discharged without sequelae. Given the risk of sudden deterioration, even initially mild presentations warrant 4 to 6 hours of hospital observation - especially in children - and a review of prioritization criteria to reduce treatment delays.

## Conclusion

All moderate and severe cases presented with hyperglycemia, and antivenom administration led to a substantial reduction in glucose levels within two hours, although values did not return to the normal range. An admission threshold of ≥ 142 mg/dL - the first proposed specifically for *T. serrulatus* - showed a strong association with severe progression, achieving 100% specificity and positive predictive value. Tachypnea, persistent vomiting, and hyperglycemia emerged as the most informative early indicators of severity. These findings suggest that admission blood glucose may serve as a practical and accessible biomarker for early risk stratification, reinforcing the need for prompt evaluation and close monitoring in pediatric scorpion envenomation.

However, this threshold should be interpreted strictly as a testable hypothesis rather than a clinical recommendation. The study had several limitations: a small sample size (n = 67; nine moderate/severe cases), single-center design, and associative methodology that prevented formal validation of glycemic cutoffs. There was no systematic comparison with other established severity markers (such as CK-MB, troponin, or inflammatory biomarkers), and not all patients underwent standardized laboratory testing. In addition, the study did not evaluate whether interventions based on the 142 mg/dL cutoff lead to improved clinical outcomes.

Given these constraints, the results highlight the potential utility of admission glucose as an early severity marker but do not support definitive cutoff definitions or recommendations for clinical practice. Larger, multicenter prospective studies are needed to assess discrimination, calibration, and clinical utility, adjust for confounding factors, and determine whether glucose-guided interventions can meaningfully improve patient outcomes.

## Availability of data and materials 

 All data generated or analyzed during this study are included in this article.
